# Mechanism of metabolic stroke and spontaneous cerebral hemorrhage in glutaric aciduria type I

**DOI:** 10.1186/2051-5960-2-13

**Published:** 2014-01-27

**Authors:** William J Zinnanti, Jelena Lazovic, Cathy Housman, David A Antonetti, David M Koeller, James R Connor, Lawrence Steinman

**Affiliations:** 1 Department of Neurology and Neurological Science, Stanford University, Stanford, CA 94305, USA; 2 Department of Radiology, University of California, Los Angeles, CA 90095, USA; 3 Department of Pathology, Penn State College of Medicine, Hershey PA, USA; 4 Department of Ophthalmology and Visual Sciences, Molecular and Integrative Physiology, University of Michigan, Ann Arbor MI, USA; 5 Departments of Pediatrics, and Molecular and Medical Genetics, Oregon Health & Science University, Portland OR, USA; 6 Department of Neurosurgery, Penn State College of Medicine, Hershey PA, USA

**Keywords:** Metabolic stroke, Glutaric aciduria, Blood–brain barrier, Cerebral hemorrhage

## Abstract

**Background:**

Metabolic stroke is the rapid onset of lasting central neurological deficit associated with decompensation of an underlying metabolic disorder. Glutaric aciduria type I (GA1) is an inherited disorder of lysine and tryptophan metabolism presenting with metabolic stroke in infancy. The clinical presentation includes bilateral striatal necrosis and spontaneous subdural and retinal hemorrhages, which has been frequently misdiagnosed as non-accidental head trauma. The mechanisms underlying metabolic stroke and spontaneous hemorrhage are poorly understood.

**Results:**

Using a mouse model of GA1, we show that metabolic stroke progresses in the opposite sequence of ischemic stroke, with initial neuronal swelling and vacuole formation leading to cerebral capillary occlusion. Focal regions of cortical followed by striatal capillaries are occluded with shunting to larger non-exchange vessels leading to early filling and dilation of deep cerebral veins. Blood–brain barrier breakdown was associated with displacement of tight-junction protein Occludin.

**Conclusion:**

Together the current findings illuminate the pathophysiology of metabolic stroke and vascular compromise in GA1, which may translate to other neurometabolic disorders presenting with stroke.

## Background

Ischemic and hemorrhagic strokes have been extensively characterized and studied [1,2]. A third type of stroke, known as metabolic stroke, begins with metabolic dysfunction and leads to a rapid onset of lasting focal brain lesions in the absence of large vessel rupture or occlusion [3-5]. The mechanism by which global metabolic dysfunction leads to focal brain injury in metabolic stroke is not well understood. Pure metabolic stroke is routinely reported in glutaric, isovaleric, methylmalonic and propionic acidurias [5]. Additionally, the organic acidurias have been associated with spontaneous intracranial hemorrhage, suggesting a vascular component may contribute to brain injury in these disorders [6,7]. The subdural and retinal hemorrhages frequently found in glutaric aciduria type I (GA1) may be mistaken for non-accidental head trauma, with severe legal and emotional consequences for families [7,8]. The etiologic role of vascular pathology in metabolic stroke has not been previously elucidated.

GA1 provides a prototypical model for metabolic stroke as more than 90% of children with this disease will experience bilateral basal ganglia injury if not identified and treated pre-symptomatically [9,10]. The disorder is caused by a deficiency of glutaryl-coenzyme-A dehydrogenase (EC 1.3.99.7; GCDH), inherited as an autosomal recessive condition [11]. GCDH is required for complete oxidation of lysine, hydroxylysine and tryptophan. Affected individuals accumulate glutaric and 3-hydroxy-glutaric acids in the brain, which are believed to play a primary role in the pathophysiology of the disease. As one of the more common inherited metabolic disorders, GA1 affects 1:30,000 to 1:100,000 children worldwide [9,12], with an increased frequency in genetically isolated populations such as Old Order Amish, Canadian Ojibway Cree natives, Irish Travelers, and native South Africans [10,13-15]. Children with GA1 typically develop normally through early infancy, but then may experience an encephalopathic crisis associated with non-specific illness between 6 and 36-months of age [11]. Prevention is critical as the encephalopathic crisis usually results in irreversible bilateral striatal injury with substantial morbidity including crippling dystonia, choreathetosis and shortened life span [11].

An animal model of GA1 encephalopathy was developed by providing GCDH-deficient (*Gcdh*^−/−^) mice with a high lysine or protein diet [16]. Recent work with this model showed age-dependent susceptibility to acute brain injury similar to human GA1 that was associated with differences in the amount of brain lysine accumulation and subsequent conversion to glutaric acid in 4-week versus 8-week old *Gcdh*^−/−^mice [17]. The young *Gcdh*^−/−^ mice suffer seizures, paralysis, hemorrhages, and death within 3-6-days of lysine or protein diet exposure, with the acute accumulation of brain glutaric acid at levels found in human autopsy cases. Encephalopathy in *Gcdh*^−/−^mice was associated with energy deprivation, detected as depletion of α - ketoglutarate (α KG), ATP and phosphocreatine [17]. Adult (> 8-week old) *Gcdh*^−/−^ mice survive after consuming the same high lysine diet, but all develop bilateral striatal necrosis after 6-weeks [16].

Since the young *Gcdh*^−/−^ mice develop hemorrhages and striatal injury similar to human GA1, this model provides the opportunity to investigate the specific vascular changes associated with an acute encephalopathic crisis. In the current study we first describe the neuropathology to guide our subsequent investigations, and then capture the evolution of the metabolic stroke including associated perfusion abnormalities. Additionally, we investigated the effect of metabolic compromise on the integrity of the blood–brain barrier (BBB), and potential changes in levels of vascular endothelial growth factor (VEGF) and hypoxia inducible factor 1-alpha (HIF-1 α). Our current findings detail the mechanism of brain injury in metabolic stroke and provide detailed evidence linking metabolic dysfunction to specific BBB abnormalities in *Gcdh*^−/−^ mice. These data are likely translational to patients with GA1 as well as other neurometabolic disorders presenting with stroke.

## Methods

### Materials

All chemicals were purchased from Sigma (St Louis, MO, USA) unless otherwise specified.

### Animals

*Gcdh*^−/−^ mice and age-matched wild type (WT) or heterozygous (*Gcdh*^−/+^) controls, both of mixed C57Bl/6 J X 129SvEv background [18], were generated from homozygotes maintained at Penn State College of Medicine (Department of Comparative Medicine) All animal experiments were reviewed and approved in accordance with IACUC research guidelines set forth by Pennsylvania State University and the Society for Neuroscience Policy on the use of animals in neuroscience research as previously described [17].

### Special diets

Diets were purchased from Harland Teklad (Indianapolis, IN, USA). The standard diet was the Harland Teklad 2018 diet, which is 18% protein and provides 1% lysine by weight. The protein diet (TD.03637) (70% casein) contains 62% protein, which is 4.7% lysine by weight. The lysine diet (TD.04412) was prepared by adding free lysine to a standard diet to achieve 4.7% total lysine. This level of lysine is not toxic in normal animals [19,20]. All special diet treated animals were evaluated daily for symptoms as previously described [16]. In order to reduce the number of animals used for these experiments, the protein diet was used for all histology experiments [16]. The lysine diet, which causes a slower onset of encephalopathy, was used for MRI experiments to avoid animals being too ill to be scanned and for long-term studies such as the BBB protein analysis.

### Neuropathology

Six 4-week old *Gcdh*^−/−^, 3 WT and 3 Gcdh−/+ mice were sacrificed before starting the diet (0 hour control). To follow the earliest pathologic events, 36 *Gcdh*^−/−^, 3 WT and 3 Gcdh−/+ mice were placed on a high protein diet at 4-weeks of age. Six *Gcdh*^−/−^mice were sacrificed every 12-hours after starting the diet and 3 WT and 3 Gcdh−/+ mice were sacrificed at 72-hours with the last 6 *Gcdh*^−/−^ mice. A second group of 36 *Gcdh*^−/−^, 6 WT and 6 Gcdh−/+ mice were placed on the lysine diet at 4-weeks of age and processed similarly. All the above mice were perfusion fixed and processed for histology and electron microscopy. Four additional *Gcdh*^−/−^ mice were immersion fixed to show *in situ* red blood cells and engorged vessels.

All mice were anesthetized with 100 mg/kg pentobarbital (i.p.), perfused with lactated Ringers (Baxter Deerfield, IL, USA) followed by 4% paraformaldehyde with 1% glutaraldehyde in 0.2 M cacodylate buffer for 15-minutes. For histology, brains were removed and post-fixed in 4% paraformaldehyde for 48-hours, and paraffin embedded. For electron microscopy, brains were removed and post-fixed for 48-hours in perfusion buffer fixative.

### Histology

H & E slides were prepared from paraffin embedded brains. Sequential 10 μm thick coronal sections were made within 0.5 mm of the Bregma line for sections including striatum and between Bregma −1.5 and −2.0 for sections including hippocampus [21].

### Electron microscopy

Brains were dissected into cortical, hippocampal or striatal blocks and embedded in Epon resin. Toluidine blue stained semithin Sections 1 μm thick were made from these blocks. Ultrathin sections of 90 nm were then cut from the same blocks and stained with uranyl acetate and lead citrate for transmission electron microscopic analysis using a Philips CM10 transmission electron microscope.

### Immunohistochemistry and confocal microscopy

Glial fibrillary acidic protein (GFAP) and occludin (Occl) were detected using deparaffinized 10 μm thick coronal brains sections. Sections were blocked with normal serum and doubled labeled with polyclonal anti-GFAP (1:500) (Dako, Carpenteria, CA, USA) and monoclonal anti-Occl (1:200) (Zymed, South San Francisco, CA, USA). Additional slides were prepared with GFAP labeling alone. All incubations were in PBS with normal serum overnight at 4°C. Double or single labeled slides were washed separately 3 times each in PBS and incubated with goat anti-rabbit IgG coupled to Cy^2^ for GFAP and goat anti-mouse IgG coupled to Cy^3^ for Occl. Polyclonal GFAP alone was detected with goat anti-rabbit coupled to horseradish peroxidase (all secondary antibodies diluted 1:2000; Jackson ImmunoResearch, West Grove, PA, USA). All slides were counterstained with 4’,6’-Diamidino-2-phenylindole (DAPI) 0.1 μg/ml in PBS for 5 minutes. Confocal microscopy was performed using a Leica TCS SP2 AOBS confocal microscope (Leica Microsystems Wetzlar, Germany).

### Capillary counts

Capillaries were counted under 10× magnification of 1 μm thick semithin sections prepared as above. Coronal sections were examined from cortex, bregma zero; hippocampus, bregma-2.30 mm; and striatum, bregma zero according to ‘The Mouse Brain in Stereotaxic Coordinates’ [21]. Total capillaries were counted within a 300 μm × 300 μm square section centered within the tissue sample. Capillaries were identified by the presence of at least one endothelial cell lining the lumen with identifiable nucleus and the correct size of 3–5 μm [22]. Occluded capillaries were counted as collapsed or with stasis, showing no patent lumen.

### Evans blue perfusion

*Gcdh*^−/−^, WT and Gcdh−/+ mice were placed on the normal or high protein diet for up to 72-hours (n = 5 per diet *Gcdh*^−/−^, n = 3 per diet WT or Gcdh−/+). Each animal was anesthetized as above and then perfused through the heart with 5% solution of Evans blue in normal saline for 30-seconds. Animals were sacrificed and brains were immersion fixed inside the skull for 48-hours in 4% paraformaldehyde. Brains were examined in whole and then sectioned under dissecting microscope. Additional control animals were used with brief (2-3-seconds) Evans blue injection followed by complete dissection to reliably differentiate arterial from venous structures. Evans blue concentration was measured in brain samples using spectrophotometry and corrected for protein content as previously described [23].

### Western blot analysis

Brain protein extracts from mice from each treatment group (20 μg each as determined by Bio-Rad protein assay, Hercules, CA, USA) were loaded on 10% sodium dodecyl sulfate-polyacrylamide gels and transferred onto nitrocellulose membranes, blocked in PBS-Tween 20 (0.1%) containing 5% non-fat dry milk and 0.1% BSA for 1 h at 4°C and incubated with monoclonal antibodies against Occludin, ZO-1 (both from Zymed, South San Francisco, CA, USA), Hypoxia inducible factor 1 alpha (HIF-1α) (RD System, Abingdon, England), vascular endothelial growth factor (VEGF) (Santa Cruz Biotechnology, Santa Cruz, CA, USA) or anti phospho-occludin at Ser 490 [24] overnight at 4°C. Blots were washed with PBS-Tween 20 (0.1%) and incubated with horseradish peroxidase conjugated anti-mouse IgG (1:10,000) for 2 h at 4°C. Immunoreactive bands were visualized using an enhanced chemiluminiscence system (ECL; Amersham Biosciences). Densitometric analysis of immunoreactive bands was performed by using ImageQuant 5.2 software (Amersham Biosciences) and results were expressed as percentage of control (WT standard diet). As a loading control we reprobed the blots with anti-actin (Sigma-Aldrich) in order to control for small variability in the gel loading.

### MRI angiography and perfusion

Magnetic resonance (MR) angiography and perfusion was performed on a 7.0 T Bruker system using a 2 mm birdcage coil. *Gcdh*−/− and WT mice were imaged at 0, 36, 72 and 96-hours following the start of lysine diet (N = 11 *Gcdh*−/−, N = 4 WT). Prior to imaging mice were anesthetized with 3% isoflurane, adjusted during the imaging to 1–1.5% in order to maintain a constant breathing rate of 40 bpm. Arterial spin labeling (ASL) was used to acquire a single-slice perfusion-weighted image at the level of the striatum (TR/TE = 2000/12 ms, NAX = 2, 1.06 mm slice thickness, 208^2^ μm^2^ in-plane resolution). A RARE sequence with multiple TR times (100-5000 ms), same slice position and resolution, was used to calculate T1 values. Perfusion values were calculated on a pixel by-pixel basis using NIH Image J (NIH; rsbweb.nih.gov/ij/) and MRI analysis calculator, a plug-in written by Karl Schmidt (NIH; rsbweb.nih.gov/ij/plugins/mri-analysis.html). MR angiography was done using a 3D gradient recalled echo sequence (TR/TE = 19/3 ms or TR/TE = 100/3 ms in order to allow slower moving blood to be visualized, 117^3^ μm^3^ voxel size, NAX = 1), and maximum intensity projection for image reconstruction.

### Statistics

Normally distributed data sets were analyzed by t-test, ANOVA or ANOVA with repeated measures with Fisher LSD or Holm-Sidak post-hoc test. Kruskal-Wallis one-way analysis of variance on ranks was performed with Student-Neuman-Keuls post hoc test on samples that were not normally distributed. Sigma Stat software (Jandel Scientific, San Rafael, CA) was used for analysis. All p-values less than 0.05 were considered statistically significant.

## Results

### Venous congestion and hemorrhage

**Figure 1:**
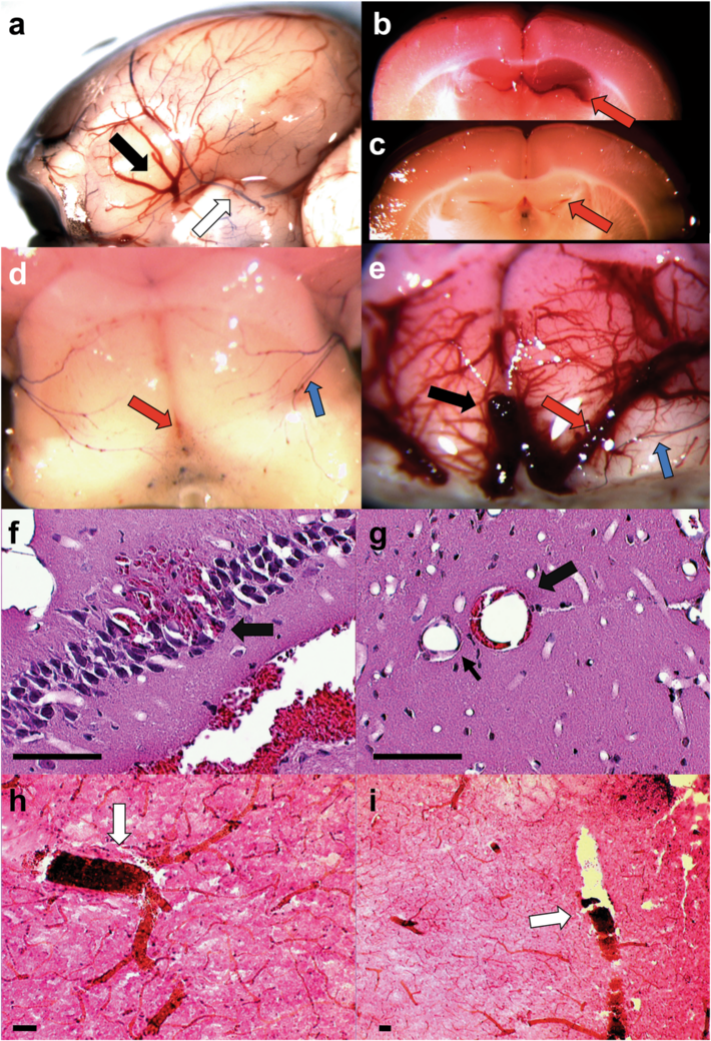
**Vessel changes associated with encephalopathy in *Gcdh***^**−/− **^**mice.** Dilation of cerebral veins noted near circle of Willis on underside of *Gcdh*^−/−^ mouse brain after protein diet exposure (**a**, black arrow). Brief Evans blue injection highlights arterial vessels of the circle of Willis (**a**, white arrow). **b** Section though cortex at Bregma −2.76 shows dilated left cerebral vein occupying entire space between hippocampus and thalamus (**b**, red arrow) with standard diet *Gcdh*^−/−^ control for comparison **(c)**. **d** and **e** shows dorsal aspect of thalamus with overlying cortex and hippocampus removed. Note extreme dilation of internal cerebral veins (**e**, red arrow) and great vein of Galen (**e**, black arrow) with normal appearing posterior cerebral artery branch marked with Evans blue injection (**e**, blue arrow). Vein of Galen is barely visible at this magnification in standard diet *Gcdh*^−/−^ control (**d**, red arrow). **f** Perfusion fixed sections of *Gcdh*^−/−^ mouse brain 36-hours after protein diet exposure shows venous congestion below hippocampus with extrusion of blood into CA3 pyramidal cell layer (arrow). **g** Perivascular collection of red blood cells outside endothelial layer (thick arrow). Note normal appearing vessel in same region (thin arrow). **h** and **i** Striatal sections of immersion fixed brain show congestion of larger “non-exchange” vessels (white arrows, venuole **h** and artery **i**). (**e-g**, Hematoxylin & eosin of perfusion fixed brain, scale bars = 200 μm).

Four-week old *Gcdh*^-/ -^mice placed on an increased lysine or protein diet were shown previously to develop hemorrhages similar to human GA1 [16]. Therefore, we first examined gross brain specimens from control and *Gcdh*^−/−^ mice placed on a standard or protein diet (60% protein) in order to identify hemorrhage locations. Brief Evans blue injection was used to differentiate arterial from venous structures. Pathologic changes were limited to *Gcdh*^−/−^ mice on the protein and lysine diet, in which venous dilation and engorgement developed within 36-48-hours. Enlarged veins were evident on the outer surface of the brain (Figure 1a). Coronal sections showed substantial enlargement of internal cerebral veins that drain the caudate/putamen bilaterally (Figure 1b and e). On further dissection between the hippocampus and thalamus, symmetric dilation of the cerebral veins coalesced into a large dilated great vein of Galen (Figure 1e). These findings are consistent with a recent report showing that brain injury in children with GA1 was also associated with enlargement of the vein of Galen [4].

Microscopic inspection of perfusion fixed brain from *Gcdh*^−/−^ mice on the protein diet showed evidence of hemorrhage (Figure 1f) and BBB compromise as red blood cells were found outside the endothelial cell layer (Figure 1g). Additional immersion fixed brain sections showed engorgement of larger non-exchange vessels (Figure 1h and i). Together these pathological changes suggest early filling of the venous system resulting in increased venous pressure, BBB breakdown and hemorrhage. Similar findings associated with venous dilation and engorgement have been reported in arteriovenous malformations where shunting of non-exchange vessels results in early filling of venous structures [25,26].

### Microscopic and ultrastructural changes

**Figure 2:**
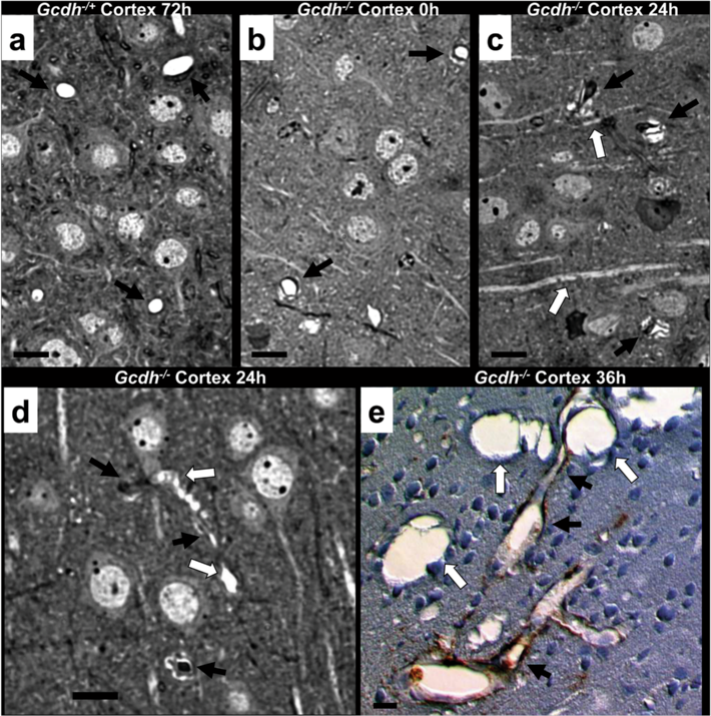
**Neuronal swelling impinges on brain capillaries.** Perfusion fixed brain sections from *Gcdh*^−/+^ control **(a)** show normal cortical neuropil and capillary morphology (black arrows). **b***Gcdh*^−/−^ mice maintained on a standard diet show small lucent regions outside capillaries (black arrows). **c** After 24-hours of protein diet, lucent regions become more pronounced (black arrows) associated with swelling and edema of cortical axons (white arrows). **d***Gcdh*^−/−^ cortex shows vacuoles filling axon of large cortical neuron (white arrows) impinging on capillaries (black arrows) with some showing stasis at 24-hours. **e** Section of cortex from *Gcdh*^−/−^ mouse 36-hours after protein diet showing large vacuoles (white arrows) impinging on blood vessel (black arrows) surrounded by astrocyte end-feet labeled with GFAP. (sections prepared by perfusion fixation, e – labeled for GFAP with DAB staining, counterstained with toluidine blue, scale bar = 10 um).

Since vessel changes were consistently found in 100% of the 4-week old *Gcdh*^−/−^mice after initiation of protein diet, we examined semi-thin sections from each of 12-hour intervals after diet exposure to capture the evolution of this process. Cortical sections from *Gcdh*^−/−^ mice on a standard diet showed slightly increased perivascular spaces around some capillaries compared to the normal morphology of *Gcdh*^−/+^ controls (compare Figure 2a and b). Perivascular spaces become more pronounced at 24-hours after protein diet exposure in *Gcdh*^−/−^ mice (Figure 2c). This change is associated with compromise of capillary lumens as some local axons are edematous. Vacuoles fill axons of large cortical neurons (Figure 2d). These findings were previously demonstrated as the initial pathologic changes after protein or lysine diet exposure in *Gcdh*^−/−^ mouse brain [17]. The local expansion of these structures impinges on capillaries with severe compromise of vessel lumens. This process is further elucidated by GFAP labeling in *Gcdh*^−/−^ cortical sections at 36-hours after protein diet exposure (Figure 2e). GFAP labeling shows intact astrocyte end-feet surrounding capillary lumens with severe compromise via locally expanding vacuoles. These vacuoles are devoid of staining indicating that they are not of astrocyte origin similar to previously reported findings [17].

**Figure 3:**
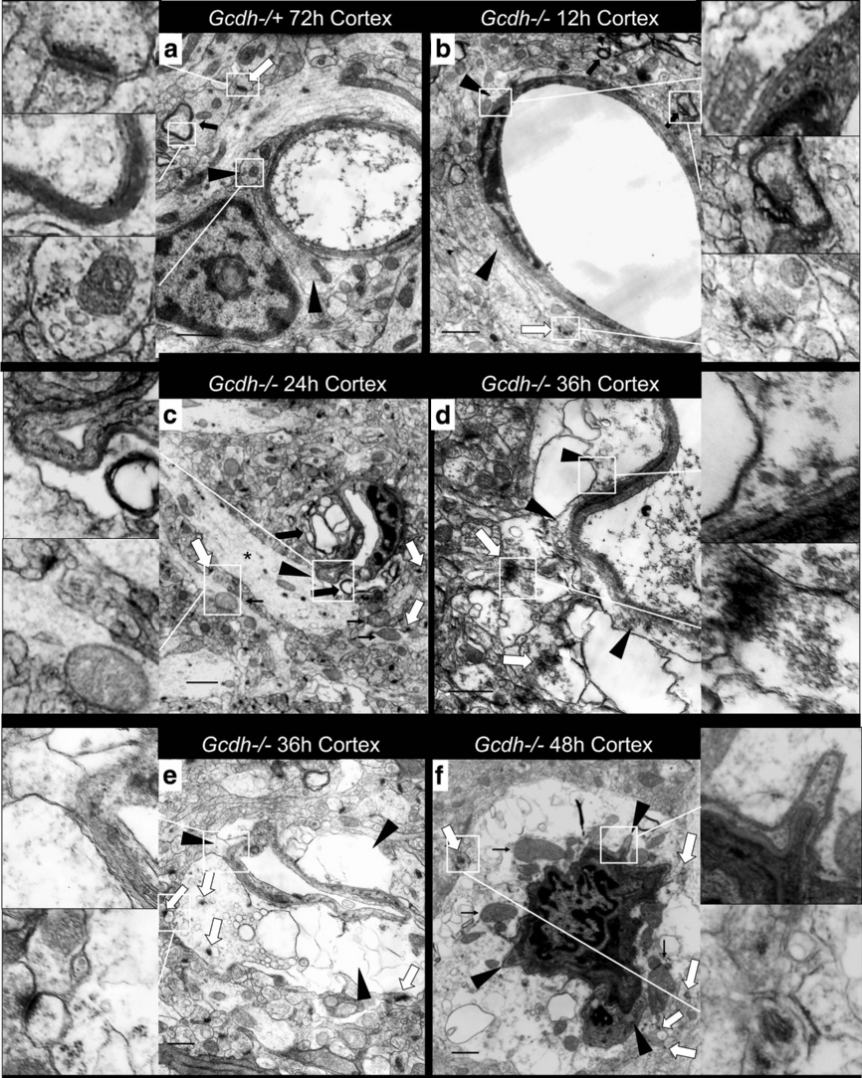
**Ultrastructural vessel changes.** Normal capillary morphology with surrounding astrocyte end-feet (arrow heads), neuronal projections, identified by synapses (white arrows), and myelinated axons (black arrows) of Gcdh−/+ **(a)** and *Gcdh*^−/−^**(b)** mouse cortex after 72 and 12-hours of protein diet exposure, respectively (see insets for enlargement of indicated sections). **c** Compressed capillary lumen surrounded by edematous myelinated axons (thick black arrows) and dendrites (white arrows) with swelling mitochondria (thin black arrows) of *Gcdh*^−/−^ mouse 24-hours after initiation of protein diet. Note intact astrocyte process with nearly normal morphology (*) touching basal lamina of capillary lumen (arrowhead). **d** Multiple edematous dendrites, identified by synapses (white arrows) and synaptic vesicles expand in proximity to capillary lumen with adjacent astrocyte end-feet (arrowheads). **e** Compressed capillary surrounded by swollen astrocyte end-feet (black arrowheads) and edematous neuronal projections identified by synapses (white arrows) both with swollen mitochondria. **f** Severely compromised capillary lumen in *Gcdh*^−/−^ mouse cortex at 48-hours of protein diet exposure surrounded by edematous dendrites identified by synapses (white arrows) and multiple swollen mitochondria (thin black arrows) with compressed astrocyte end-feet (black arrowheads). (Perfusion fixed for EM, scale bar = 1 um).

We next used electron microscopy to inspect ultrastructural changes around brain capillaries at different time points after protein diet exposure (Figure 3). Multiple capillary lumina in *Gcdh*^−/−^ mouse brain were compromised with increasing severity between 24 and 48-hours after protein diet exposure (Figure 3c-f). Gcdh−/+ and *Gcdh*^−/−^ cortex after 72 and 12-hours of protein exposure respectively show normal vessel morphology (Figure 3a and b). In contrast, after 24-hours of protein diet, *Gcdh*^−/−^ cortical capillaries showed compressed lumens impinged by edematous axons and neuronal projections with swollen mitochondria (Figure 3c). Higher magnification of similar sections showed increasing edema of dendrites in local neuropil with swelling of astrocyte end-feet after 36-hours (Figure 3d). At 36-hours after protein diet exposure some cortical capillaries show both intact and swollen astrocyte end-feet with severely swollen local neuronal projections and further compromise of capillary lumina (Figure 3e). Swollen astrocyte end-feet are suggestive of post-ischemic changes within 5-hours of ischemic event [27]. After 48-hours of protein diet exposure we observed capillaries that were severely compromised with surrounding expanded neuronal projections, identified by synapses with extensive mitochondrial swelling (Figure 3f). Together these findings confirm our previous observation that neuronal swelling is the initial pathological change in *Gcdh*^−/−^ mice exposed to a high protein diet [17]. In addition we show that neuronal swelling appears to compromise local capillary lumina.

### Ischemic changes in different brain regions

Compromised capillaries were found with variable frequency at different time points in *Gcdh*^−/−^ mouse brains after protein diet exposure. In order to characterize the pattern and timing of these changes, multiple sections of each brain region were examined for the status of capillary lumina at different time points. Table 1 shows the proportion of totally occluded or collapsed vessels in cortex, striatum and hippocampus at different time points (see methods). In the cortex more than 75% of total capillaries were occluded after 24-hours of protein diet exposure (Table 1), however, less than 50% were occluded at 36-hours, and less than 15% at 48-hours. In hippocampal sections, less than 30% of total capillaries were occluded at 24-hours, followed by an average of 80% occlusion at 36-hours and less than 50% at 48-hours (Table 1). In the striatum, there were no occluded capillaries until 36-hours of protein diet exposure when 50% were occluded, followed by 90% occluded at 48-hours (Table 1). This pattern shows that early transient cortical ischemia is followed by more severe ischemia in the striatum.

**Table 1:** Percent occluded capillary vessels

	**12**-**hour**	**24**-**hour**	**36**-**hour**	**48**-**hour**
**Cortex**	0% ± 5%	75% ± 10%	45% ± 5%	12% ± 2%
**Hippocampus**	0% ± 5%	25% ± 5%	80% ± 15%	40% ± 10%
**Striatum**	0% ± 5%	0% ± 5%	50% ± 8%	90% ± 10%

Semi-thin sections prepared at 12, 24, 36 and 48-hours after initiation of protein diet showed substantial occlusion of capillary vessels in cortex, hippocampus and striatum of *Gcdh*^−/−^ but not Gcdh−/+ control brain. (n = 6 per time point, all changes significant p < 0.001 compared to control).

**Figure 4:**
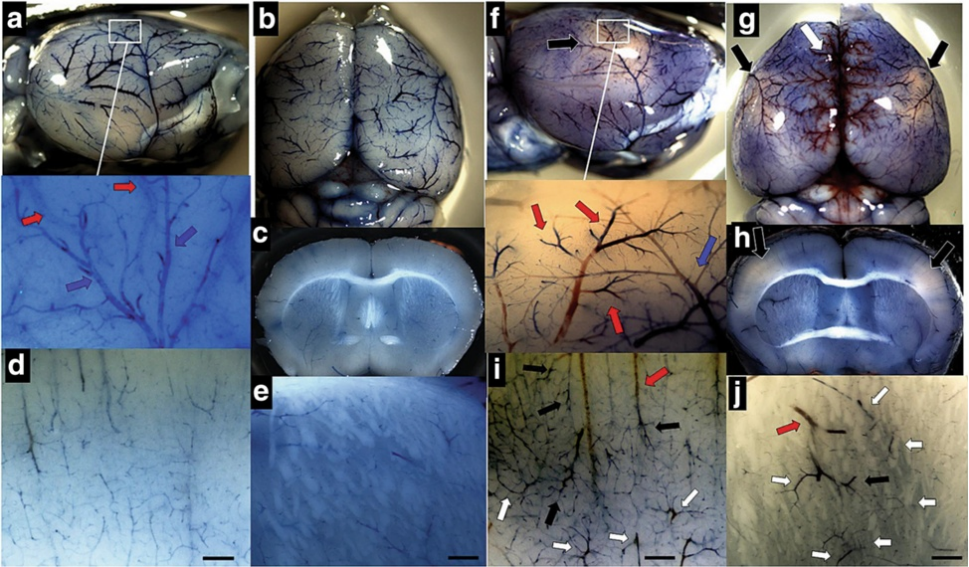
**Bilateral ischemia and vessel changes in *Gcdh***^**−/− **^**mouse brain after protein diet exposure.** Comparison of *Gcdh*^−/+^ and *Gcdh*^−/−^ whole brain profile **(a and f)** with magnified cortical surface (inset), dorsal surface **(b and g)** and coronal section **(c and h)** with magnified areas of cortex **(d and i)** and striatum **(e and j)** both placed on the protein diet for 72 and 36-hours respectively. Evans blue injection shows normal vessel morphology with complete and smooth filling of cerebral vessels in *Gcdh*^−/+^ mouse. Inset **(a)** shows magnification of middle cerebral artery branch (blue arrow) and pial veins (red arrows). Ischemia is noted as lack of blue staining (black arrows) in profile **(f)** and bilaterally on dorsal view **(g)** and coronal section **(h)** of *Gcdh*^−/−^ mouse. Inset **(f)** shows incomplete filling of middle cerebral artery branch (blue arrow) and differential filling of pial vein branches (red arrows). Magnified areas of cortex and striatum **(i and j)** show prominence of non-exchange vessels (>10 μm, white arrows) venous congestion (red arrows) and differential filling of return vessels (black arrows). (Evans blue protocol detailed in methods, scale bar = 100 μm).

The quantity and severity of occluded vessels at different time points observed by histology suggested a pattern of ischemia that is likely detectable grossly in whole brain. In order to capture these changes, Evans blue was injected through the heart at different times in *Gcdh*^−/−^ and *Gcdh*^−/+^ control mice on the high protein diet. Figure 4 shows a repeating pattern of ischemia at 36-hours after protein diet exposure in *Gcdh*^−/−^ mice, which consistently showed pale ischemic regions of cortex and striatum bilaterally (Figure 4f-h). These ischemic regions are supplied via end branches of the middle cerebral artery in each hemisphere. On closer inspection (Figure 4f inset) evidence of shunting is found as venous end branches are filling before arterial end branches. A similar pattern is found in the cortex and striatum where non-exchange vessels (larger than 10 μm) become prominent (Figure 4i and j). Larger venules show congestion with differential filling of end branches and surrounding areas of pale cortex indicating lack of capillary bed filling (Figure 4i and j).

### Compromised capillary bed perfusion and increased blood–brain barrier permeability

**Figure 5:**
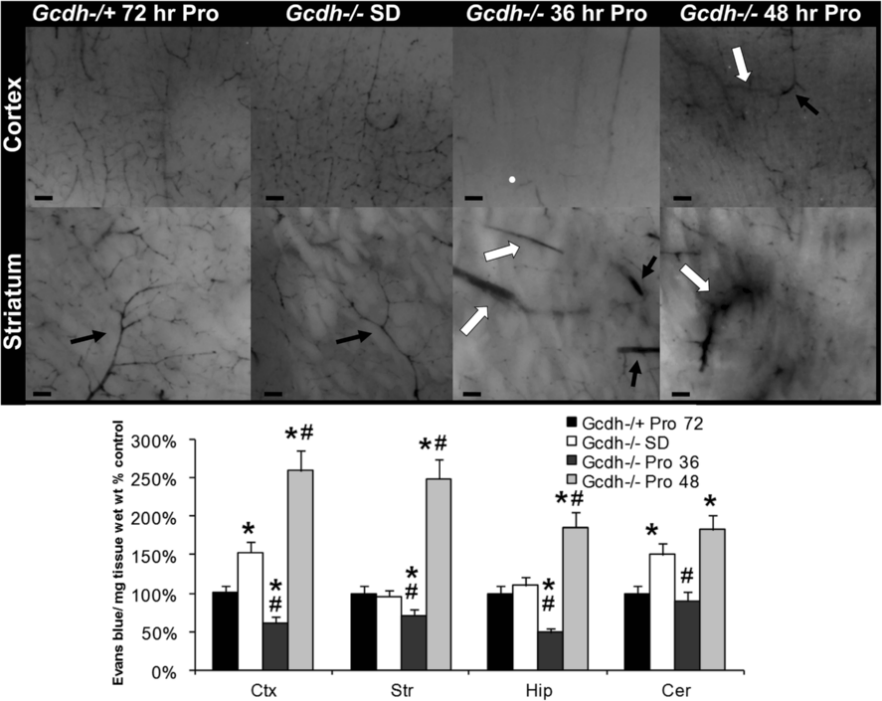
**Microscopic perfusion changes.** Evans blue perfusion shows normal capillary bed network in cortex (top row) and striatum (bottom row) of Gcdh−/+ control after 72-hours of protein diet and *Gcdh*^−/−^ mice on standard diet. Note normal appearance of striatal arteries (black arrows). After 36-hours of protein diet, *Gcdh*^−/−^ mice show loss of capillary bed perfusion with continued filling of larger non-exchange vessels. Note dilated striatal arteries (black arrows) and veins (white arrows). After 48-hours of protein diet exposure, capillary bed perfusion is limited with prominence of larger non-exchange vessels. Note continuity between non-exchange vessels in cortex (arrows) and increased background signal indicating permeability (scale bars = 200 μm). Bar graph) *Gcdh*^−/−^ mouse brain samples from cortex (Ctx), striatum (Str), hippocampus (Hip) and cerebellum (Cer) were evaluated for Evans blue concentration after standard (SD) or high protein (Pro) diet compared to *Gcdh*^−/+^ control. (± S.E.M, * p < 0.05 compared to *Gcdh*^−/+^, # p < 0.05 compared to *Gcdh*^−/−^ SD, n = 4 per group).

We next used microscopic examination of Evans blue perfused brains to localize vascular changes associated with ischemic brain regions. Here we show loss of capillary bed perfusion within 36-hours of protein diet exposure in *Gcdh*^−/−^ mouse cortex and striatum (Figure 5). Larger non-exchange vessels are shown with continued filling and enlargement especially in striatum (Figure 5, lower panel). Capillary bed perfusion continues to be severely limited at 48-hours in *Gcdh*^−/−^ brain (Figure 5, far right), and background staining around vessels indicates increased blood–brain barrier permeability. Additionally, continuity found between larger non-exchange vessels is consistent with shunting and early filling of the venous system found on inspection of whole brain (Figure 4).

In order to quantify these changes, we measured Evans blue concentration in perfused brain sections spectrophotometrically. Evans blue content did not vary significantly between *Gcdh*^−/+^ controls with and without the protein diet. Therefore, concentrations are reported as percentage of control after 72-hours of protein diet exposure (Figure 5, bar graph). Both *Gcdh*^−/−^ cortex and cerebellum showed increased signal compared to control before protein diet exposure. This difference is likely associated with an increased baseline permeability of *Gcdh*^−/−^ brain vessels. *Gcdh*^−/−^ cortex, striatum and hippocampus all showed decreased signal compared to control consistent with occluded vessels and lack of capillary bed filling in *Gcdh*^−/−^ brain 36-hours after protein diet exposure (Figure 5). After 48 hours of protein exposure, increased signal was found in all brain sections compared to control, consistent with increased BBB permeability after initial ischemia in these brain regions.

### MRI angiography and perfusion

**Figure 6:**
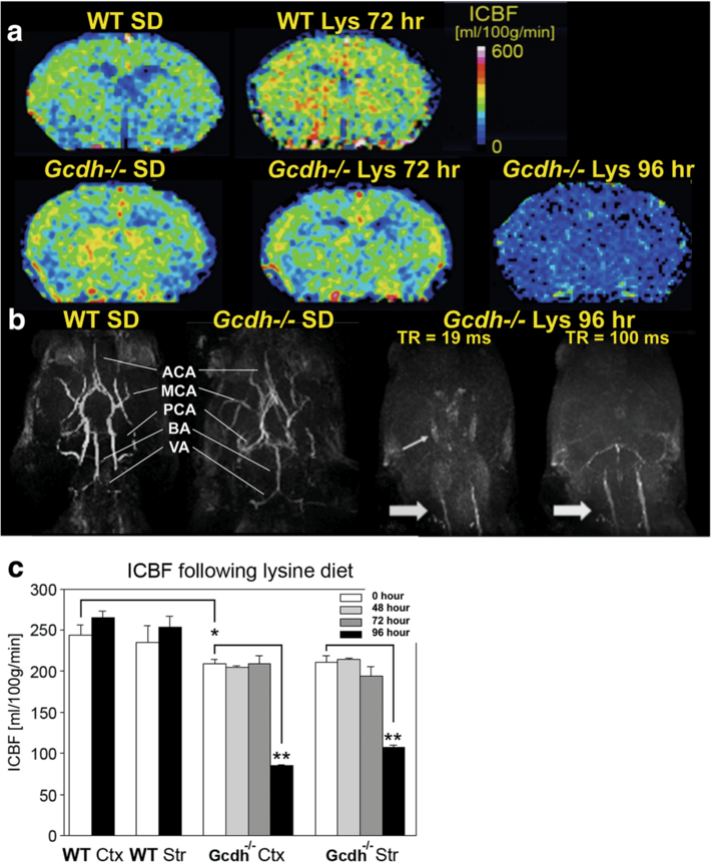
**Magnetic resonance angiography and perfusion.***Gcdh*^−/−^ and WT mice underwent MR perfusion-weighted imaging and angiography before and after lysine diet exposure. **a** Representative images showing index of cerebral blood flow (ICBF) as colorimetric scale (0–600 ml/100 mg tissue/ min). After 72-hours of lysine diet exposure, *Gcdh*^−/−^ mouse shows reduced ICBF bilaterally in the striatum (ICBF < 300 ml/100 mg tissue/min). After 96-hours, *Gcdh*^−/−^ mouse has globally reduced ICBF (< 200 ml/100 mg tissue/min). **b** Normal visualization of circle of Willis, with vertebral (VA), basilar (BA), posterior (PCA), middle (MCA) and anterior (ACA) cerebral arteries from bottom up in WT and *Gcdh*^−/−^ mouse on a standard diet (SD). Complete loss of signal in *Gcdh*^−/−^ mouse brain is noted after 96-hours of lysine diet using standard acquisition time (19 ms). Note signal from carotid arteries (large arrow) and circle of Willis (small arrow). Using 5-fold longer acquisition time (100 ms) a small area of the posterior circulation is visible just above the carotid arteries (large arrow). **c** Quantitative ICBF changes were significant in cortical and striatal ICBF in WT (n = 4) and *Gcdh*^−/−^ mice (n = 5) following lysine diet exposure (* p < 0.05 t-test, **p < 0.05 ANOVA with repeated measures).

Based on the perfusion changes detected with Evans blue injection, we reasoned that these differences may be detectable with MR perfusion-weighted imaging and MR angiography *in vivo*. Figure 6a and c shows relative quantification of perfusion as index of cerebral blood flow (ICBF) using a colorimetric scale. In WT mice there was a slight increase in ICBF after 72-hours of lysine diet in both cortex and striatum although not significant. *Gcdh*^−/−^ mice on a standard diet had significantly reduced cortical ICBF compared to WT mice on a standard diet (Figure 6c). After 96-hours of lysine diet exposure there was a substantial decrease in ICBF both in the cortex and in the striatum of *Gcdh*^−/−^ mouse by 50% compared to standard diet control (Figure 6a and c).

MR angiography showed reduced blood flow as a complete loss of signal from cerebral vessels between 72-96-hours after lysine diet exposure in *Gcdh*−/− mice. Figure 6b shows representative MR angiogram in WT and *Gcdh*^−/−^ mice on a standard diet, and *Gcdh*^−/−^ mouse after 96-hours of lysine diet. When an extended acquisition time (TR = 100 ms) was used to maximize the inflow signal and allow for slower blood flow to be visualized, only the posterior circulation including basilar and posterior cerebral arteries were identified (Figure 6b, far right). Taken together these results suggest a bilateral, frontal greater than posterior deficit in cerebral arterial flow associated with lysine diet exposure in *Gcdh*^−/−^ mice. These findings are consistent with our Evans blue studies and previous findings in human GA1 showing decreased perfusion during encephalopathic crisis [28].

### Molecular basis of blood–brain barrier weakness

**Figure 7:**
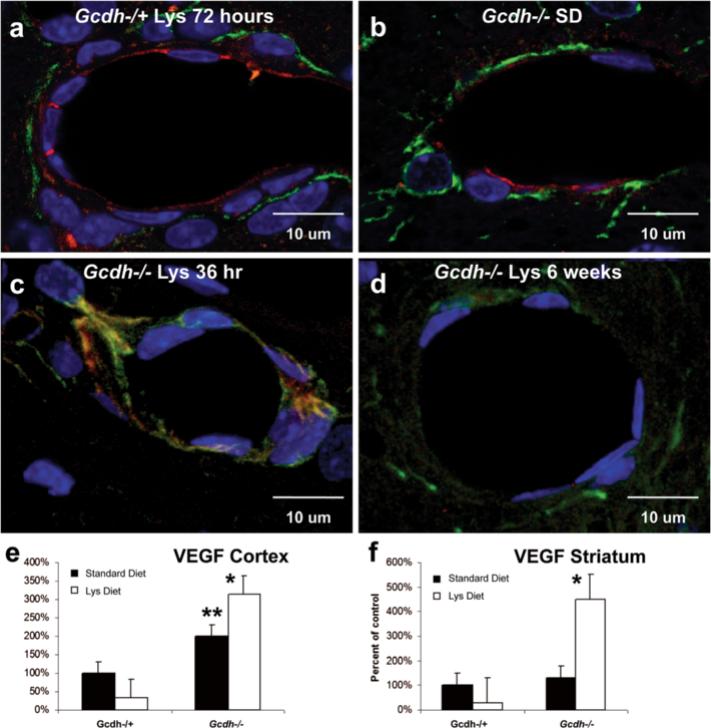
**Confocal microscopy of blood**–**brain barrier.** Cortical sections of perfusion fixed brain from *Gcdh*^−/−^ and heterozygous (*Gcdh*^−/+^) mice were fluorescently labeled for astrocytes with GFAP (green), occludin (red) and nuclei with DAPI (blue). **a***Gcdh*^−/+^ mouse cortex shows astrocyte end-feet (green) outlining larger blood vessel with complete ring of occludin between endothelial cells representing intact blood brain barrier. **b** Similar section of *Gcdh*^−/−^ mouse on a standard diet (SD) showing incomplete ring of occludin at blood–brain barrier. **c** Within 36-hours of lysine diet exposure in the *Gcdh*^−/−^ mouse, occludin is no longer between endothelial cells and appears to be withdrawn from the vessel lumen. **d** After 6-weeks of lysine diet exposure in *Gcdh*^−/−^ mice, signal from occludin is not detectable at blood–brain barrier. **e** and **f** VEGF was significantly increased in cortex of *Gcdh*^−/−^ mice compared to control and further increased after 36-hours of high lysine diet in cortex **(e)** and striatum **(f)**. (± S.E.M, ** p < 0.05 compared to standard diet control, * p < 0.02, n = 3-4 per group).

In the current study we have shown increased cerebral capillary permeability in *Gcdh*^−/−^ mice consistent with previous findings [16]. We hypothesized that these pathological features may be associated with an intrinsic weakness in tight-junction proteins that form and maintain the BBB [29,30]. Therefore, we tested the localization and relative concentration of tight-junction proteins occludin and ZO-1 in *Gcdh*^−/−^ mice and *Gcdh*^−/+^ control. Here we show confocal microscopy evidence of BBB disruption after lysine diet exposure in *Gcdh*^−/−^ mice (Figure 7). Double labeled sections of *Gcdh*^−/+^ controls show astrocyte end-feet (GFAP) extending to the cerebral vessels with complete localization of occludin between endothelial cells of the BBB (Figure 7a). In *Gcdh*^−/−^ mouse brain, incomplete occludin labeling is found at the BBB on a standard diet for all brain sections examined (cortex, striatum and hippocampus) (Figure 7b). After 36-hours of lysine diet exposure, occludin is shown withdrawn from the cell borders in *Gcdh*^−/−^ mouse brain (Figure 7c). In *Gcdh*^−/−^ mice that survive the lysine diet and remain on the diet for 6 weeks, complete loss of signal from occludin is shown (Figure 7d). These changes were consistent in all brain sections examined and show intrinsic weakness of the BBB in *Gcdh*^−/−^ mice that is exacerbated with lysine diet exposure. This weakness is shown to be associated with loss of tight-junction protein occludin at the BBB. Similar changes were not found for ZO-1 (Additional file 1: Figure S1).

Recent work by Murakami and colleagues [31] demonstrates a central role for occludin phosphorylation and trafficking away from the BBB in VEGF mediated BBB permeability. Based on the disruption of occludin localization at the BBB in *Gcdh*^−/−^ mice, we investigated both VEGF levels and phosphorylation status of occludin in control and *Gcdh*^−/−^ mice with and without lysine diet (Figure 7 and Additional file 1: Figure S1). VEGF, at baseline, was significantly increased in the cortex of *Gcdh*^−/−^ mice compared to *Gcdh*^−/+^ control (Figure 7). After 36-hours of lysine diet exposure VEGF was further increased in the cortex. Preliminary results showed an increase in the phosphorylation status of occludin compared to control (Additional file 1: Figure S1). VEGF was also found to be elevated in the striatum after 36-hours, providing additional support for a role of VEGF in the abnormal localization of occludin (Figure 7) and increased vascular permeability (Figure 5). These data are consistent with incomplete occludin localization at the BBB in *Gcdh*^−/−^ mice and previous studies that showed increased expression of VEGF in *Gcdh*^−/−^ mouse brain [32]. Testing for changes in hypoxia inducible factor 1 alpha (HIF1α) were inconclusive (data not shown). Increased VEGF has previously been associated with ischemic neurons [33], and is consistent with the evidence of tissue ischemia presented in Table 1 and Figures 5 and 6. Together these data suggest a mechanism of induced BBB weakness and permeability in GA1.

## Discussion

**Figure 8:**
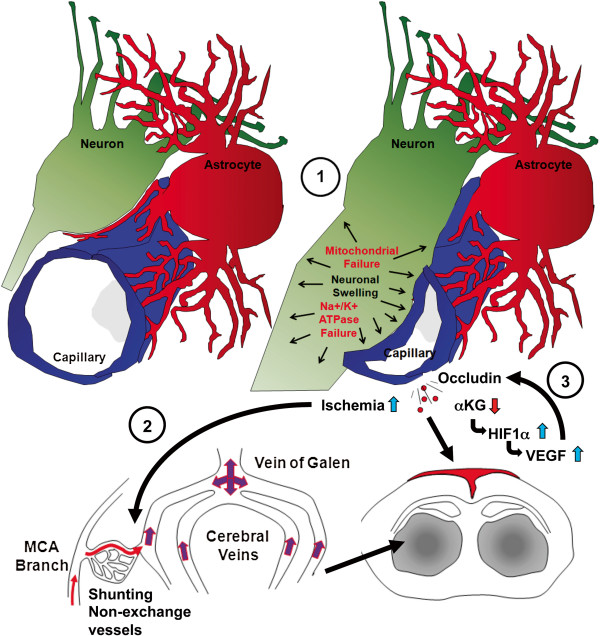
**Mechanism of metabolic stroke.** Metabolic dysfunction associated with glutaric acid production and accumulation results in mitochondrial energy failure with secondary failure of Na/K ATPases and edema initially of neurons and neuronal projections (**1**). Neuronal expansion impinges on capillary blood vessels leading to ischemia, which compounds and expands regions of neuronal swelling. Compression of capillaries leads to shunting of blood to non-exchange vessels with early filling and dilation of the deep venous system (**2**). Lack of valves in cerebral veins allows for symmetric decreased flow from striatum and thalamus. Chronic metabolic dysfunction depletes α KG levels leading to lack of HIF1a degradation and up regulation of VEGF leading to vessel expansion and weakness including mobilization of tight-junction proteins away from blood–brain barrier (**3**). The combination of vessel impingement, shunting and weakened blood–brain barrier likely results in hemorrhages.

In this work we present several lines of evidence to show that brain injury in GA1 involves a metabolic stroke, the proposed mechanism of which is diagramed in Figure 8. In contrast to ischemic stroke, metabolic stroke begins with neuronal swelling associated with mitochondrial failure. Neuronal vacuolation and swelling in cortex and striatum was previously shown as the first neuropathological change in *Gcdh*^−/−^ mice [17]. Here we show that swelling of neuronal projections impinges on local capillary vessels causing ischemia. Similar to ischemic stroke, swelling of astrocyte end-feet after initial ischemia was shown to further compromise capillary lumens [27]. Compromised capillary perfusion leads to shunting of blood flow through non-exchange vessels with early filling of the venous system (Figure 8). These findings parallel perfusion changes and cerebral vessel enlargement, found in human GA1 that predicted shunting of blood to non-exchange vessels [4,28]. Early filling of the deep brain venous system leads to expansion of these thin walled vessels lacking support of a muscular media [34]. Similar findings have been reported for arterial venous malformation of the basal ganglia where early filling of the venous system was shown to cause dramatic expansion to these vessels [25,26]. The lack of valves in the cerebral venous system provides an even distribution of symmetric expansion of these structures (Figure 8). Combined with BBB weakness, increased pressure associated with shunting and early filling of the venous system likely accounts for hemorrhages in GA1.

Cerebrovascular weakness has been frequently reported in GA1 as subdural and retinal hemorrhages, previously misdiagnosed as non-accidental head trauma [8,10]. Similarly, the GA1 mouse model has consistently shown development of intracranial hemorrhages associated with encephalopathic crisis when exposed to protein or lysine diets that raise brain glutaric and 3-OH-glutaric acid levels [16]. Previous *in vitro* studies have shown increased permeability of striatal but not cortical rat brain endothelial cell monolayers exposed to glutaric and 3-OH-glutaric acids [16]. Similar work by Muhlhausen and colleagues showed disruption of human derived endothelial cells exposed to 3-OH-glutaric acid [35]. The mechanism underlying a specific BBB weakness in GA1, however, has been elusive. Initial evidence that VEGF may play a role in these vascular anomalies came from microarray studies examining expression patterns in *Gcdh*^−/−^ mouse brain that showed increased expression of VEGF [32]. Recent data by Murakami and coworkers demonstrates the pivotal role of VEGF in mobilization of the tight-junction protein occludin away from the BBB [31]. These data are consistent with our current findings that VEGF is increased and occludin is partially disrupted at baseline in the brain of *Gcdh*^−/−^ mice and may account as a possible mechanism of BBB weakness in GA1.

Recent studies have shown that the interaction between HIF1 α and VEGF can be modulated by the availability of α KG [36]. In light of our previous findings that α KG levels are depleted in the brain of *Gcdh*^−/−^ mice, we can now propose a mechanism to explain how metabolic dysfunction causes specific BBB weakness in GA1 [17]. As diagramed in Figure 8, production of glutaric acid in the brain is associated with depletion of α KG, which correlates with decreased glutamate and γ -aminobutyric acid (GABA) levels that are dependent on available α KG [17,37]. Decreased α KG also leads to increased HIF1 α [36], which induces VEGF, already increased at baseline and exacerbated further during encephalopathy. VEGF stimulates phosphorylation and mobilization of occludin, which allows for increased permeability and loss of integrity of the BBB [31]. In the context of a failing BBB, the increased pressure associated with shunting to non-exchange vessels during encephalopathy likely accounts for hemorrhages in GA1 [4,28].

Striatal injury is the hallmark of neuropathology in GA1 and other disorders presenting with metabolic stroke [5,38,39]. Extrastriatal neuropathology involving the cortical grey and white matter are also reported, but less recognized and studied [40,41]. Recent work by Harting and colleagues follows MRI changes in pre-symptomatic GA1 patients and shows delay in early frontotemporal cortical development that is already present at birth [42]. In the majority of treated patients, this frontotemporal underdevelopment was shown to normalize by 4-years of age [42]. In contrast, patients that experience encephalopathy are more likely to show frontotemporal atrophy and spongiform degeneration of white matter tracts [40-42]. Our current findings also show cortical involvement and parallel findings in GA1 patients include evidence of decreased perfusion and glucose uptake in frontocortical regions [4,28,40]. The GA1 mouse model may provide the opportunity to further study and elucidate specific differences between cortical and striatal susceptibility.

In both mouse and human GA1 encephalopathy, the cortex shows transient and more progressive degeneration while the striatum is injured more rapidly and completely [42]. Similar findings were shown to be associated with the brain regions most affected by BBB breakdown in models of transient middle cerebral artery occlusion as well as metabolic inhibition [43,44]. Striatal degeneration is the most consistent neuropathological finding in GA1 [42], however, our current findings and other models of ischemic and metabolic stroke show that cortical involvement typically precedes and exacerbates striatal damage [44,45]. Indeed, striatal injury can be alleviated by removal of cortical projections [45-47]. Consistent with our current and previous findings, cortical neurons are more likely to recover as striatal neurons are further compromised paralleling the level of BBB damage [16,48,49]. Striatal neurons are especially at risk because of the high concentration of N-methyl-D-aspartate (NMDA) receptors and cortico-striatal glutamatergic projections as well as specific BBB vulnerability [16,50]. Previous studies have shown the selective vulnerability of the striatum to uncontrolled excitatory amino acid exposure causing excitotoxicity [51]. We and others have shown the specific vulnerability of the striatal BBB to metabolic and toxic insult as well as transient ischemic stroke [16,44,49]. Taken together, cortical insult and BBB compromise could account for selective striatal vulnerability. BBB compromise, as seen in this model, allows increased permeability of excitatory amino acids, which can lead to both acute and chronic neuronal injury with progressive degeneration seen in both mouse and human GA1. This mechanism could be in part responsible for bilateral striatal necrosis that develops over 6-weeks in adult *Gcdh*^−/−^ mice surviving on a high lysine diet where BBB occludin is undetectable [16].

## Conclusions

In the current study we have characterized metabolic stroke in a mouse model of GA1 showing initial neuronal swelling with secondary ischemia associated with impingement of brain capillaries. Capillary occlusion leads to shunting of blood to non-exchange vessels with early filling and dilation of the venous system. Loss of the tight-junction protein, occludin, is shown as an intrinsic weakness of the BBB exacerbated by metabolic encephalopathy. Together these findings may account for the intracranial hemorrhages frequently encountered in *Gcdh*^−/−^ mice, and GA1 patients, and suggest potential new targets for preventive strategies. Previous work by Antonetti and colleagues showed hydrocortisone treatment of endothelial cells *in vitro* was associated with increased occludin localization at tight-junction barriers and decreased permeability [52]. These findings suggest a potential role for hydrocortisone treatment to improve BBB integrity in GA1.

## Competing interests

The authors declare that they have no competing interests regarding data presented in this manuscript.

## Authors’ contributions

Experiments were done by WZ, JL, CH, and DA. Data were analyzed by WZ, JL, CH, DA, DK, JC and LS. Paper was written by WZ, JL, DA, DK, JC and LS. All authors read and approved the final manuscript.

## Supplementary Material

Additional file 1: Figure S1 Brain tissue Western blot analysis. Representative western blot analysis for brain protein extracts from *Gcdh*^−/−^ and wild type (WT) mice placed on the lysine (Lys) diet for 36-hours. Phosphorylated Occludin data tabulated at lower right. (n = 3-4 samples per group, *p < 0.05 compared to WT). Click here for file
