# Supplementary figures and images for: Mechanism of metabolic stroke and spontaneous cerebral hemorrhage in glutaric aciduria type I
Source: Acta Neuropathol Commun. 2014 Jan 27;2:13. doi: 10.1186/2051-5960-2-13 (PMC3940023; doi:10.1186/2051-5960-2-13)

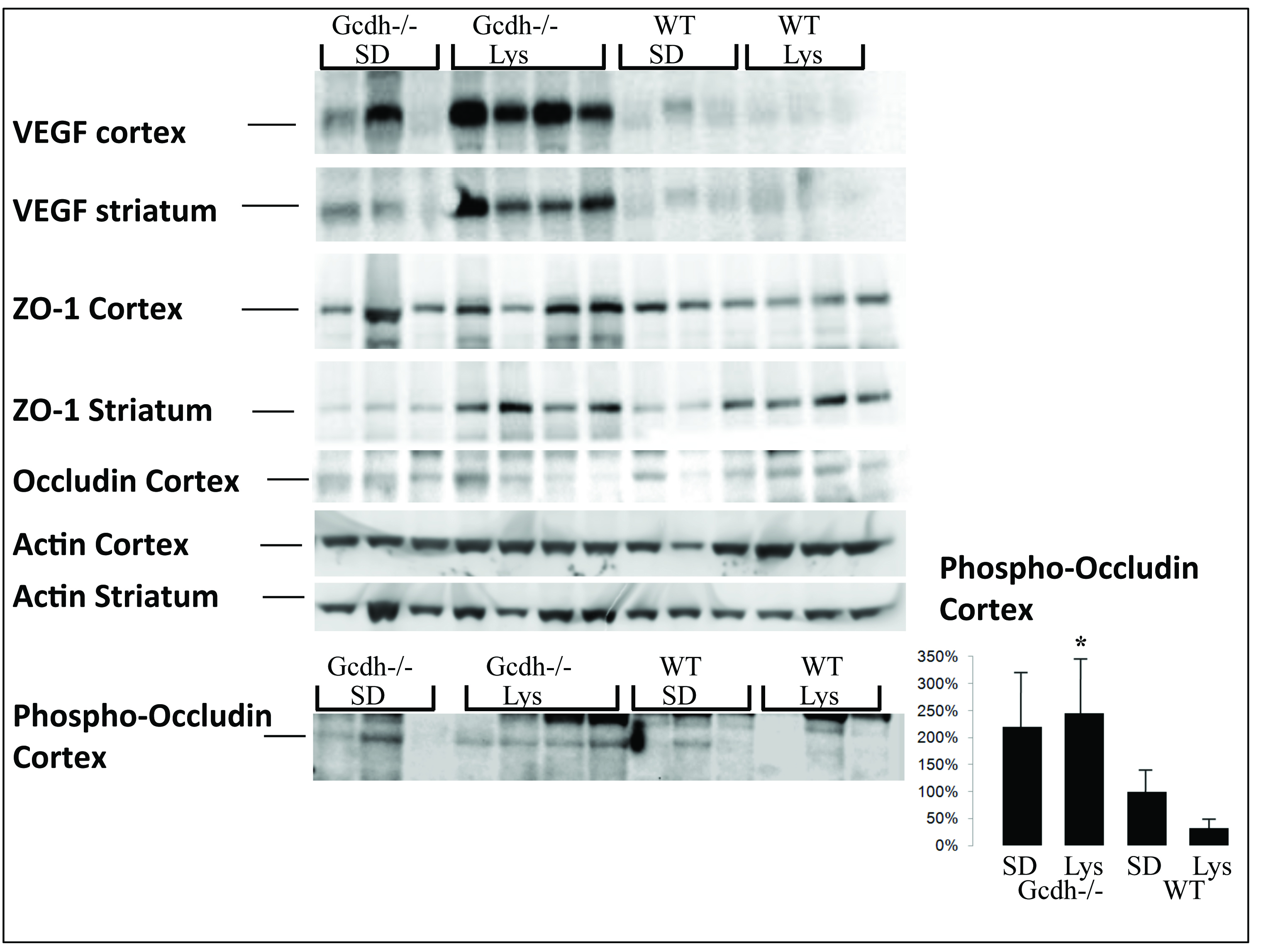

Supplement: Additional file 1: Figure S1 — Brain tissue Western blot analysis. Representative western blot analysis for brain protein extracts from Gcdh−/− and wild type (WT) mice placed on the lysine (Lys) diet for 36-hours. Phosphorylated Occludin data tabulated at lower right. (n = 3-4 samples per group, *p < 0.05 compared to WT). [file 2051-5960-2-13-S1.jpeg]
